# Proteomic Analysis of Roots Response to Potassium Deficiency and the Effect of TaHAK1-4A on K^+^ Uptake in Wheat

**DOI:** 10.3390/ijms232113504

**Published:** 2022-11-04

**Authors:** Ke Xu, Yong Zhao, Yaxin Yu, Ruoxi Sun, Weiwei Wang, Shuhua Zhang, Xueju Yang

**Affiliations:** 1 State Key Laboratory of North China Crop Improvement and Regulation, North China Key Laboratory for Crop Germplasm Resources of Education Ministry, Key Laboratory for Crop Germplasm Resources of Hebei, Hebei Agricultural University, Baoding 071000, China; 2 Cangzhou Academy of Agriculture and Forestry Sciences, Cangzhou 061001, China

**Keywords:** wheat, potassium (K^+^), tandem mass tag (TMT), TaHAK1-4A, K^+^ uptake

## Abstract

Potassium (K^+^) is essential for plant growth and stress responses. A deficiency in soil K^+^ contents can result in decreased wheat quality and productivity. Thus, clarifying the molecular mechanism underlying wheat responses to low-K^+^ (LK) stress is critical. In this study, a tandem mass tag (TMT)-based quantitative proteomic analysis was performed to investigate the differentially abundant proteins (DAPs) in roots of the LK-tolerant wheat cultivar “KN9204” at the seedling stage after exposure to LK stress. A total of 104 DAPs were identified in the LK-treated roots. The DAPs related to carbohydrate and energy metabolism, transport, stress responses and defense, and post-translational modifications under LK conditions were highlighted. We identified a high-affinity potassium transporter (TaHAK1-4A) that was significantly up-regulated after the LK treatment. Additionally, *TaHAK1-4A* was mainly expressed in roots, and the encoded protein was localized in the plasma membrane. The complementation assay in yeast suggested that TaHAK1-4A mediates K^+^ uptake under extreme LK conditions. The overexpression of *TaHAK1-4A* increased the fresh weight and root length of Arabidopsis under LK conditions and improved the growth of Arabidopsis *athak5* mutant seedlings, which grow poorly under LK conditions. Moreover, silencing of *TaHAK1-4A* in wheat roots treated with LK stress decreased the root length, dry weight, K^+^ concentration, and K^+^ influx. Accordingly, TaHAK1-4A is important for the uptake of K^+^ by roots exposed to LK stress. Our results reveal the protein metabolic changes in wheat induced by LK stress. Furthermore, we identified a candidate gene potentially relevant for developing wheat lines with increased K^+^ use efficiency.

## 1. Introduction

Potassium (K^+^) is an essential macronutrient that directly or indirectly affects plant physiological and biochemical processes crucial to growth, development, quality, and yield [1,2]. Additionally, K^+^ can increase the resistance of crop plants to biotic and abiotic stresses (e.g., cold, waterlogging, drought, salinity, and sodicity) [3].

As a major staple food consumed by billions of people, wheat (*Triticum aestivum* L.) provides humans with a large proportion of the daily intake of nutrients [4]. In terms of agricultural production, a stable and sufficient supply of soluble K^+^ fertilizer is important for maintaining wheat quality and yield. An exposure to K-deficient conditions results in obvious phenotypic changes in wheat plants (e.g., wilting, leaf chlorosis from the tip to the edge, and ultimately necrosis) [1]. Although soil reserves of K^+^ are generally large, most soil K^+^ (90–98%) is in a structural form, such as feldspar and mica, and only a limited amount of soil K^+^ is in bioavailable water-soluble (0.1–0.2%) and exchangeable (1–2%) forms [5,6]. Therefore, large amounts of chemical K^+^ fertilizers are applied annually, thereby increasing farming costs and the potential risk of environmental pollution [7]. There is thus an urgent need to improve how K^+^ fertilizers are absorbed and used by plants to minimize their application. The identification of key candidate genes is the basis for characterizing the molecular mechanisms underlying K^+^ absorption and use.

The development of large-scale sequencing technology has provided researchers with another tool for studying K^+^ absorption and utilization. We previously conducted an integrated analysis of gene expression and metabolite profiles that revealed the importance of genes related to ion homeostasis, cellular reactive oxygen species (ROS) homeostasis, and the glutamate metabolic pathway in response to K-deficiency [8]. Zhang et al. [9] identified 258 peptides in cotton (*Gossypium* spp.) xylem sap, while also confirming that low-K^+^ (LK) stress significantly alters the abundance of environmental stress-related proteins, decreasing plant tolerance to external stresses. In another study, an iTRAQ-based proteomics approach was used to characterize the proteome profiles of alligator weed (*Alternanthera philoxeroides* L.) under LK conditions; a pathway enrichment analysis indicated that most of the differentially abundant proteins (DAPs) were related to carbohydrate and energy metabolism, secondary metabolism, signal sensing and transduction, stress responses, and protein synthesis or degradation [10]. However, fully elucidating the LK response mechanism of wheat will require considerably more research.

In response to soil K^+^ deficiency, crops have evolved multiple strategies to meet the demand for K^+^ during growth, which include altering the morphological structure of the root system, enhancing K^+^ absorbance, and reallocating cytosolic K^+^ to maintain cellular homeostasis [11]. In plants, K^+^ absorption and distribution are mediated by potassium channels and transporters. The HAK/KUP/KT (high-affinity K^+^/K^+^ uptake/K^+^ transporter) family is the largest potassium transporter family in plants [12,13]. Genes encoding HAK/KUP/KT transporters are ubiquitous among plants, with 13 in Arabidopsis (*Arabidopsis thaliana*), 16 in peach (*Prunus persica* L. Batch), and 27 in barley (*Hordeum vulgare* L.), rice (*Oryza sativa* L.), and maize (*Zea mays* L.) [14,15,16,17]. The HAK/KUP/KT gene family comprises four major clusters (I–IV), of which several members of cluster I have been thoroughly characterized. For example, the expression of *OsHAK1*/*5*/*16*, *AtHAK5*, *HvHAK1*, and *ZmHAK1/5* is induced under LK conditions, with the encoded proteins helping to maintain K^+^ uptake or translocation in plants [18,19,20,21,22,23]. Moreover, AtHAK5 is a high-affinity K^+^ transporter that facilitates K^+^ uptake under severely K^+^-deficient conditions; *athak5* mutants exhibit inhibited root growth only in response to LK stress [24,25,26]. The *HvHAK1* gene, which is the closest homolog of *AtHAK5* in barley, also contributes to K^+^ uptake [22]. In rice, *OsHAK1* and *OsHAK5,* which are highly similar to *AtHAK5* (i.e., sequence identity), encode high-affinity K^+^ transporters [13] that differentially affect K^+^ distribution and rice architecture, with OsHAK1 mediating K^+^ acquisition and translocation at both low and high K^+^ concentrations, whereas OsHAK5 is functional only under LK conditions [19,20]. According to these earlier studies, HAK transporters vary in terms of their functions related to K^+^ uptake or translocation. Cheng et al. [12] identified 56 putative HAK/KUP/KT family members in wheat (reference: TGACv1) and determined that TaHAK1b-2BL mediates K^+^ transport in yeast. However, additional research conducted to clarify the role of HAK transporters in plants, especially in common wheat, is needed.

In this study, the root proteome profile of the LK-tolerant cultivar “KN9204” exposed to K-deficient conditions was analyzed using a tandem mass tag (TMT)-based comparative proteomics approach. Moreover, the importance of the high-affinity K^+^ transporter TaHAK1-4A during wheat responses to LK stress was verified in yeast, Arabidopsis, and wheat. This investigation of protein metabolic networks in wheat provides new insights into the mechanisms underlying wheat tolerance to LK stress and may form the foundation for future genetic improvements.

## 2. Results

### 2.1. Wheat Root Proteomic Profiles under LK Conditions

**Figure 1:**
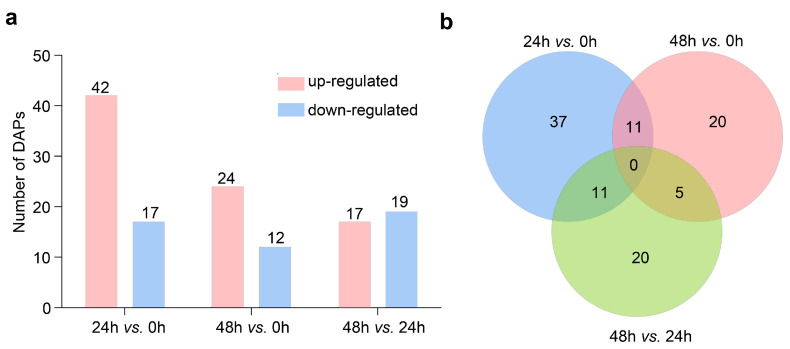
Differential abundance proteins (DAPs) responsive to low-K^+^ (LK) stress in wheat roots. (**a**) Number of DAPs in different comparisons. (**b**) The Venn diagram describing overlaps among DAPs in different comparisons. Twelve-day-old seedlings under full K^+^ condition (6 mM K^+^) were subjected to LK stress under modified Hoagland solution containing LK (15 µM K^+^) and the roots harvested at 0, 24, and 48 h.

In our TMT-based proteomic analysis, we identified 50,522 peptides from 123,107 peptide-spectrum matches, as well as 9535 proteins, of which 9527 were common in the 9 examined root samples. Specific details regarding these proteins are listed in Table S1. We used |fold change| > 1.20 and *p* < 0.05 as the thresholds for assessing the significance of LK-induced changes to protein contents, which resulted in the identification of 104 significant DAPs (Table S2). More specifically, 59 (42 up-regulated/17 down-regulated), 36 (24 up-regulated/12 down-regulated), and 36 (17 up-regulated/19 down-regulated) DAPs were detected in the 24 h vs. 0 h, 48 h vs. 0 h, and 48 h vs. 24 h comparisons, respectively (Figure 1a). The Venn diagram indicated that 37, 20, and 20 of the DAPs were specifically up-regulated or down-regulated in the 24 h vs. 0 h, 48 h vs. 0 h, and 48 h vs. 24 h comparisons, respectively (Figure 1b). In addition, the changes in the abundances of 11 DAPs were similar (8 up-regulated and 3 down-regulated) in the 24 h vs. 0 h and 48 h vs. 0 h comparisons (Table S2). These DAPs included a tonoplast intrinsic protein (TIP; W5G5H0) and heat shock protein 90 (HSP90; F4Y590). It is considered that HAKs are directly involved in potassium absorption or transport. Notably, the abundance of a potassium transporter (A0A3B6I5B5) increased significantly (fold change of 1.23) after 24 h exposure to LK stress.

### 2.2. Functional Analysis of DAPs Related to LK Stress Responses

**Figure 2:**
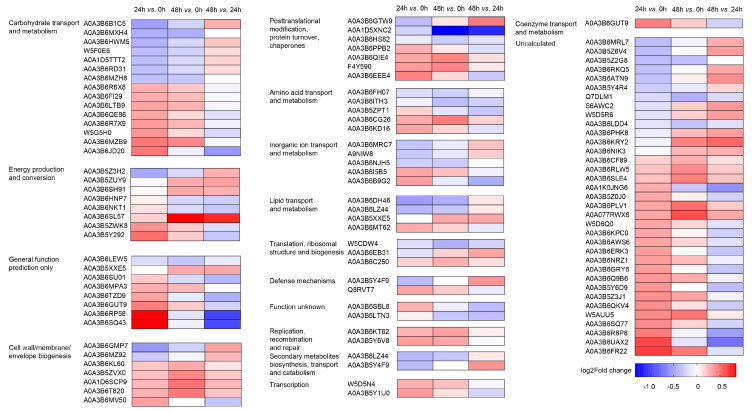
Function clusters of Orthologous Groups of Protein (COG) classification for the differential abundance proteins (DAPs) in wheat roots under LK stress.

The DAPs in the LK-stressed wheat roots were mainly assigned to 16 COG categories (Figure 2). Of the DAPs annotated according to the COG database, most were associated with “carbohydrate transport and metabolism” (15 DAPs), “energy production and conversion” (8 DAPs), “cell wall/membrane/envelope biogenesis” (7 DAPs), “posttranslational modification, protein turnover, chaperones” (7 DAPs), “inorganic ion transport and metabolism” (5 DAPs), and “amino acid transport and metabolism” (5 DAPs).

**Figure 3:**
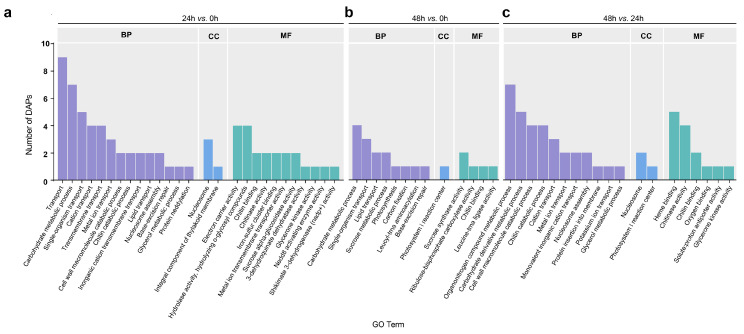
Gene Ontology (GO) analysis of low-K^+^ responsive differential abundance proteins (DAPs) in roots of wheat. (**a**–**c**) The GO in 24 h vs. 0 h, 48 h vs. 0 h, and 48 h vs. 24 h comparisons, respectively. BP: biological process; CC, cellular component; MF: molecular function.

A GO analysis was performed to functionally annotate the 104 DAPs. The broad range of GO terms assigned to the DAPs included 23, 3, and 17 terms from the biological process, cellular component, and molecular function categories, respectively (Figure 3). Three carbohydrate-related GO terms (“carbohydrate metabolic process”, “carbohydrate derivative metabolic process”, and “carbon fixation”) were assigned to 15 DAPs (e.g., sucrose synthase and ATP synthase subunit alpha). Eleven DAPs (e.g., potassium transporter, putative zinc transporter, multidrug resistance protein 1 homolog, HMA domain-containing protein, tonoplast intrinsic protein) were annotated with transport-related GO terms, including “potassium ion transport”, “transport”, “metal ion transport”, and “transmembrane transport”.

### 2.3. Protein–Protein Interaction (PPI) Network Analysis of the DAPs Related to LK Stress Responses

**Figure 4:**
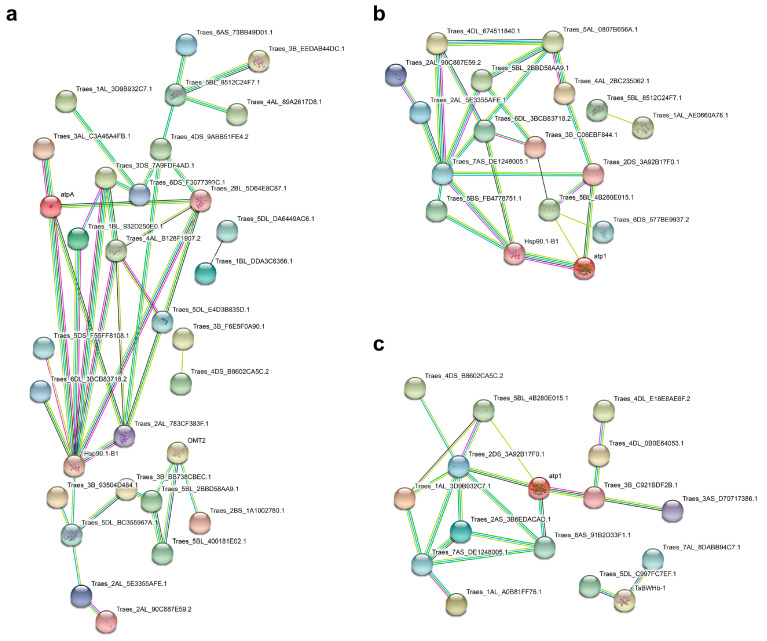
The protein–protein interaction (PPI) network of differential abundance proteins (DAPs) in LK stressed roots. (**a**–**c**) The PPIs in 24 h vs. 0 h, 48 h vs. 0 h, and 48 h vs. 24 h comparisons, respectively.

To reveal the complex network of interacting proteins associated with wheat root responses to potassium deprivation, a PPI network was predicted using the String database. The corresponding relationships between protein_IDs and PPI map gene_IDs are listed in Table S3. A total of 31, 17, and 16 DAPs were included in the PPI map for the 24 h vs. 0 h, 48 h vs. 0 h, and 48 h vs. 24 h comparisons, respectively (Figure 4). In the 24 h vs. 0 h comparison, Hsp90.1-B1, Traes_2BL_5D64E8C87.1, Traes_2AL_783CF383F.1, and Traes_4AL_B128F1907.2 were the proteins with the most interactions. In the 48 h vs. 0 h comparison, Traes_7AS_DE1248005.1 and Traes_6DL_3BCB83718.2 were the two major proteins that interacted with other proteins. The PPI network for the DAPs in the 48 h vs. 24 h comparison suggested Traes_2DS_3A92B17F0.1 and Traes_7AS_DE1248005.1 may be involved in regulating wheat root responses to potassium deficiency.

### 2.4. Expression Analysis and Molecular Characterization of TaHAK1-4A

**Figure 5:**
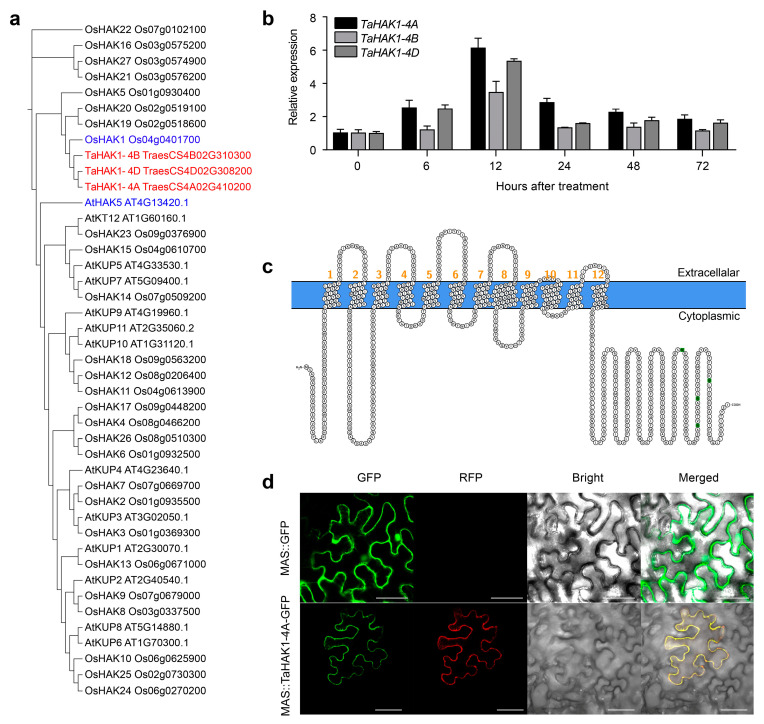
Characteristics and expression of TaHAK1-4A. (**a**) Phylogenetic tree of TaHAK1-4A, OsHAKs, and AtHAKs. Ta, *Triticum aestivum*; Os, *Oryza sativa*; At, *Arabidopsis thaliana*. (**b**) Expression analysis of TaHAK1s at transcript level. Twelve-day-old seedlings at two-leaf stage were treated with LK stress for the indicated durations. (**c**) Predicted transmembrane segments of TaHAK1-4A. (**d**) Subcellular location of TaHAK1-4A in *Nicotiana benthamiana* leaves. Scale bars = 50 µm.

According to our TMT data, the abundance of a potassium transporter (A0A3B6I5B5 encoded by *TraesCS4A02G410200*) increased after the exposure to LK stress (Table S2). The phylogenetic analysis of *TraesCS4A02G410200, TraesCS4B02G310300*, and *TraesCS4D02G308200* (i.e., three gene copies on chromosomes 4A, 4B, and 4D, respectively) and HAK-encoding rice genes indicated these three wheat genes are closely related to *OsHAK1*. We named *TraesCS4A02G410200, TraesCS4B02G310300*, and *TraesCS4D02G308200* as *TaHAK1-4A*, *TaHAK1-4B*, and *TaHAK1-4D*, respectively, according to their subgenome positions (Figure 5a).

To further elucidate TaHAK functions during the wheat LK stress response, we analyzed the expression levels of 89 *TaHAK* family members in wheat (reference genome: IWGSC v 2.0) under LK stress conditions using our published RNA-seq data [8]. The results showed that *TaHAK1-4A*, *TaHAK1-4B*, and *TaHAK1-4D* were the three *TaHAK* genes with the highest expression levels under LK conditions among the *TaHAK* genes in the wheat genome (Figure S1a). The fold changes in the expression levels of these three genes were higher in the LK-tolerant cultivar “KN9204” than in the LK-sensitive cultivar “BN207”. Additionally, *TaHAK1-4A* had the highest expression-level fold change in “KN9204” (9.72-fold), implying TaHAK1-4A is important for the LK tolerance of wheat (Figure S1b). Our analysis of the tissue-specific expression of *TaHAK1-4A* on the basis of public RNA-seq data [27] indicated *TaHAK1-4A* was predominantly expressed in wheat roots (Figure S2). The RT-PCR data confirmed that *TaHAK1-4A* expression in wheat roots was induced under LK conditions (Figure 5b). Thus, the TaHAK1-4A function was analyzed further.

### 2.5. Subcellular Localization of TaHAK1-4A

The 12 transmembrane segments revealed by Protter implied TaHAK1-4A is a transmembrane protein (Figure 5c). During the subcellular localization analysis, the fluorescence of the green fluorescent protein (GFP) alone (i.e., positive control) was detected in the cytoplasm and nucleus, whereas the TaHAK1-4A-GFP signal was exclusive to the plasma membrane (PM). Moreover, in *Nicotiana benthamiana* cells with TaHAK1-4A-GFP and the mCherry-labeled PM marker, we detected a substantial overlap in the green and red fluorescent signals (Figure 5d). Accordingly, TaHAK1-4A appears to be a PM protein in cells.

### 2.6. TaHAK1-4A Mediates K^+^ Uptake in Yeast

**Figure 6:**
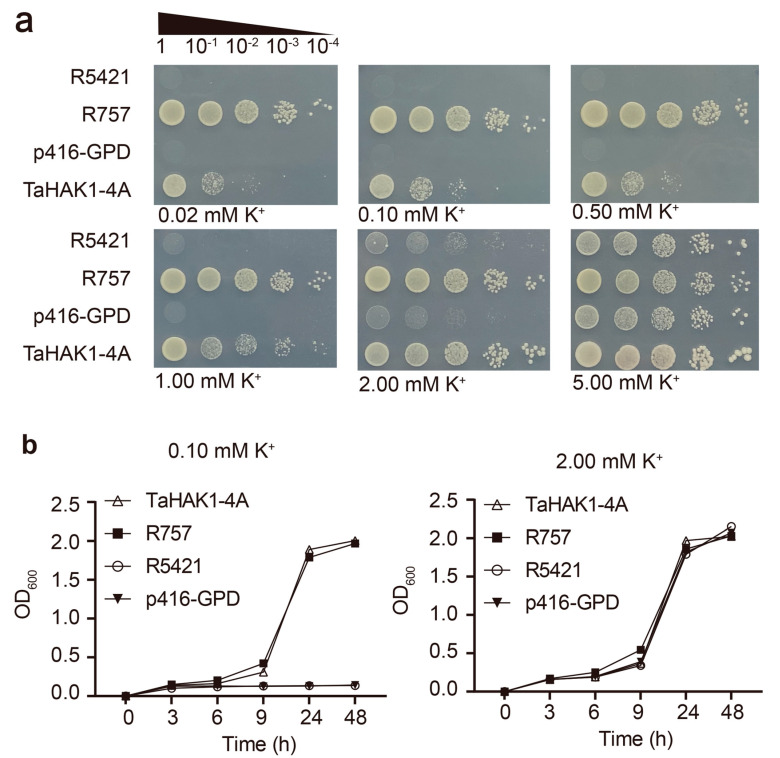
TaHAK1-4A mediates K^+^ transport in yeast. (**a**) TaHAK1-4A complement the K^+^ uptake-deficient yeast mutant R5421 on AP medium containing various K^+^ concentrations. R757 was used as positive control. (**b**) Growth curves of the R757, R5421, and R5421 strains transformed with P416-GPD (empty vector) or TaHAK1-4A in liquid AP medium with 0.10 mM K^+^ or 2.00 mM K^+^.

The R5421 yeast strain, which is defective in terms of K^+^ uptake, cannot grow under LK conditions [28]. Thus, we transformed this yeast mutant with *TaHAK1-4A* and compared its growth with R757 (i.e., the wild-type (WT) strain of R5421) to determine whether TaHAK1-4A is involved in the uptake of K^+^ (Figure 6). When the K^+^ concentration was less than 2 mM, R5421 was unable to grow. Additionally, there were no major differences among transformants when they were grown on AP medium containing 5 mM K^+^. However, TaHAK1-4A restored the growth of R5421, even under extreme LK conditions (0.02 mM K^+^). The growth curves of the yeast cells in liquid AP medium supplemented with 0.10 mM and 2 mM K^+^ further demonstrated the restorative effects of TaHAK1-4A on the growth of the transformed mutant strain. At 0.10 mM K^+^, the *TaHAK1-4A*-expressing strains and R757 grew well, with an OD_600_ of up to 2.0 at 48 h. In contrast, the OD_600_ values of the p416-GPD and R5421 strains were still below 0.2 after 48 h. At 2 mM K^+^, there were no significant growth differences among these strains, and the OD_600_ reached up to almost 2.0 within 24 h. Hence, TaHAK1-4A is a high-affinity K^+^ transporter that mediates K^+^ uptake.

### 2.7. TaHAK1-4A-Overexpressing Arabidopsis Lines Are Tolerant to LK Stress

**Figure 7:**
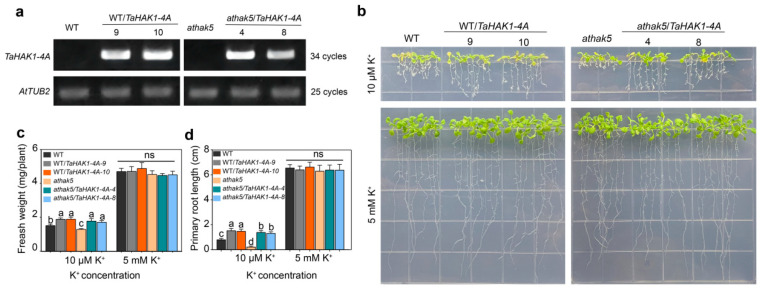
Overexpression of *TaHAK1-4A* enhanced the LK tolerance of Arabidopsis and improved the growth of *athak5* under LK condition. (**a**) Detection of *TaHAK1-4A* mRNA expression levels in Arabidopsis using demi-quantitative RT-PCR. (**b**) The phenotype analysis of Arabidopsis with various amounts of K^+^ added. (**c**) Fresh weight and (**d**) primary root length of plants in (**b**). Letters denote significantly different groups identified by Student’s *t*-test (*p* < 0.05); ns indicates non-substantial differences at that level of significance.

In the phylogenetic tree, TaHAK1-4A was most closely related to AtHAK5 in Arabidopsis (Figure 5a). To functionally characterize TaHAK1-4A and determine whether TaHAK1-4A and AtHAK5 have similar roles, WT Arabidopsis and the *athak5* mutant were transformed with *TaHAK1-4A*. In the current study, the *athak5* mutant exhibited defective growth under LK conditions, with shorter primary roots than the WT plants, but the growth could be gradually restored by increasing the K^+^ content. To confirm *TaHAK1-4A* was present and highly expressed in the transformants, two transformed WT and mutant lines underwent a semi-quantitative RT-PCR analysis (Figure 7a).

Following the 5 mM K^+^ treatment, there were no phenotypic differences between the WT and *TaHAK1-4A*-overexpressing (OE) lines, whereas the *TaHAK1-4A*-OE lines grew better than the WT plants under LK conditions (10 µM K^+^) (Figure 7b). The fresh weight and primary root length were similar between the OE lines and the WT control after the 5 mM K^+^ treatment, but the OE lines had a higher fresh weight and primary root length under LK conditions (10 µM K^+^) (Figure 7c,d). The *athak5* mutant exhibited severely defective growth in the medium supplemented with 10 µM K^+^, but the expression of *TaHAK1-4A* clearly restored the growth of the mutant to almost WT levels. The phenotypes of the *athak5* mutant and the *TaHAK1-4A*-OE lines were confirmed on the basis of the fresh weight and primary root length (Figure 7c,d). Thus, TaHAK1-4A functioned as a high-affinity K^+^ transporter in Arabidopsis exposed to LK stress.

### 2.8. TaHAK1-4A Promotes K^+^ Uptake in Wheat

**Figure 8:**
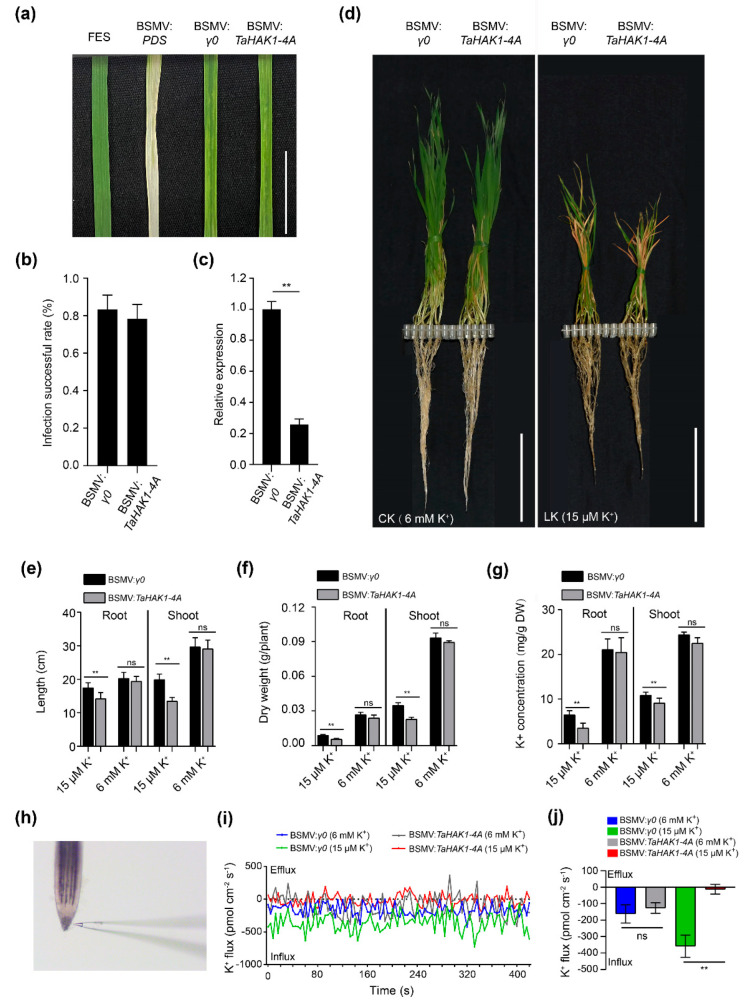
Effects of silencing *TaHAK1-4A* using VIGS technique under LK stress in wheat. (**a**) The third leaf of BSMV-inoculated plants, bar = 1 cm. (**b**) Infection success rate of BSMV-inoculated plants. (**c**) Relative expression of plants infected with BSMV:*γ0* or BSMV:*TaHAK1-4A*. (**d**) Performance of the wheat plants under control (6 mM K^+^) and LK stress (15 µM K^+^), bar = 10 cm. (**e**) Length, (**f**) dry weight, and (**g**) K^+^ concentration of plants under CK or LK conditions. (**h**) Bright-field microscopy images of the net K^+^ flux determination at root tip surface. (**i**,**j**) The rates of net K^+^ fluxes in tested materials. Error bars represent standard errors of at least three independent replicates. Student’s *t*-test was used to test the statistical significance (** *p* < 0.01) between the control and treatment.

To further clarify the molecular basis of the effects of TaHAK1-4A, we constructed BSMV-VIGS vectors to silence *TaHAK1-4A* expression in wheat. At 12 d post-inoculation, the third leaf of the wheat plants inoculated with BSMV:*PDS* (positive control) exhibited photobleaching symptoms (Figure 8a), implying our VIGS conditions were appropriate. Additionally, the plants inoculated with BSMV:*γ0* or BSMV:*TaHAK1-4A* had mild chlorotic mosaic symptoms (Figure 8a), and the efficiency of the BSMV-VIGS exceeded 78.3% (Figure 8b). Compared with the control plants, the *TaHAK1-4A* transcript levels were markedly lower in the silenced plants according to the RT-PCR results (Figure 8c). At 6 mM K^+^, there were no obvious phenotypic differences between the BSMV:*TaHAK1-4A* plants and the BSMV:*γ0* plants (control). However, compared with the BSMV:*γ0* plants, the growth of the BSMV:*TaHAK1-4A* plants was substantially inhibited (Figure 8d) under LK conditions (15 µM K^+^). In addition, the wheat root and shoot lengths, dry weights, and K^+^ concentrations of the BSMV:*TaHAK1-4A* plants decreased significantly under LK conditions (Figure 8e–g).

Non-invasive micro-test technology (NMT) was applied to compare the K^+^ flux rate in the root tips of the BSMV:*γ0* and BSMV:*TaHAK1-4A* plants (Figure 8h–j). In response to 6 mM and 15 µM K^+^, the overall K^+^ influx rate was higher for the BSMV:*γ0* plants than for the BSMV:*TaHAK1-4A* plants. Compared with the effects of 6 mM K^+^, the 15 µM K^+^ treatment resulted in a greater difference in the K^+^ influx rate between the BSMV:*γ0* and BSMV:*TaHAK1-4A* plants. The down-regulated expression of *TaHAK1-4A* decreased the influx of K^+^ into wheat roots, which indicated that the silencing of *TaHAK1-4A* impairs the ability of wheat roots to take up K^+^ from the soil.

## 3. Discussion

### 3.1. Proteins Related to Carbohydrate and Energy Metabolism in Wheat Roots under LK Conditions

Carbohydrate and energy metabolism generally changes rapidly in plants exposed to adverse environmental conditions to satisfy energy demands [29,30,31]. The transcription of genes encoding key enzymes involved in carbohydrate metabolism, including sorbitol dehydrogenase (SDH) and sucrose synthase (SUS), increases significantly in plants under K-deficient conditions [32,33,34]. In the current study, the expression of two SUS-encoding genes was up-regulated by the LK stress. Moreover, DAPs annotated as β-glucosidases and sucrose:sucrose fructosyltransferases (SSTs) increased in abundance under LK conditions. Similarly to sucrose, fructan is an important storage carbohydrate that is also involved in stress responses, partly because it is soluble and osmotically active [35,36]. A previous study demonstrated that SST is a key enzyme for fructan biosynthesis in higher plants; the overexpression of *SST* genes improves the tolerance of cotton to drought [37] and the resistance of rice to low temperatures [38]. In the present study, the expression of genes encoding enzymes associated with carbon metabolism in the roots increased after exposure to LK stress, suggesting metabolic activities are enhanced to meet the increased energy demands for K^+^ uptake and growth in wheat.

### 3.2. Proteins Related to Stress Responses and Defense in Wheat Roots under LK Conditions

Pathogenesis-related (PR) proteins, such as chitinases, peroxidases, β-1,3-glucanases, and nonspecific lipid-transfer proteins (nsLTPs), are activated in response to various biotic and abiotic stresses [39,40]. The accumulation of ROS in roots, which occurs during the early plant response to the LK stress, results in the increased mobilization of ROS scavengers, including peroxidases that can help to reinforce cell walls by catalyzing lignification-related reactions [41] and provide protection against multiple pathogens [39]. The production of three peroxidases was significantly induced in response to the LK treatment. The accumulation of these peroxidases can protect cells against membrane peroxidation and enhance the cell wall strength in wheat roots exposed to the LK stress. Chitinases are important hydrolytic enzymes that are activated by a variety of biotic and abiotic stress conditions, as well as by various phytohormones, including ethylene, jasmonic acid, and salicylic acid [42]. The results of the current study indicated that two chitinases with the highest fold changes were up-regulated under LK conditions. In addition, the production of other PR proteins, such as β-glucanases and nsLTPs, was also up-regulated. Therefore, we speculated that LK stress induces the expression of genes encoding PR proteins associated with the LK resistance of “KN9204”.

Heat shock proteins are crucial for minimizing the effects of adverse environmental stimuli because they limit protein misfolding and protein aggregation [43]. For example, the overexpression of *AtHSP90.2*, *AtHSP90.5*, and *AtHSP90.7* reportedly increases the sensitivity of Arabidopsis to salt and drought conditions [44]. Earlier research confirmed that plant SHSP20s modulate the resistance to various abiotic stresses (e.g., heat, drought, and cold) and also influence somatic embryogenesis and seed germination [45,46,47]. In the present study, HSP90 and two SHSPs (HSP20s) were up-regulated in response to LK stress. The accumulation of HSPs has also been observed in other plants exposed to LK conditions, including banana (*Musa acuminata* L.) and alligator weed [48,49]. These results suggest HSPs may participate in plant responses to LK stress. Furthermore, a LEA_2-domain-containing protein and cold-induced proteins also accumulated significantly following the LK treatments. Hence, these stress-responsive proteins may cooperatively protect plants against LK stress.

### 3.3. Proteins Related to Post-Translational Modifications in Wheat Roots under LK Conditions

Phosphorylations catalyzed by protein kinases and ubiquitinations catalyzed by ubiquitin ligases are common post-translational modifications that affect various processes in eukaryotic cells [50]. Previous studies demonstrated the regulatory effects of phosphorylation on some K^+^ transporters/channels. For example, CIPK23 can activate AtHAK5 via phosphorylation, leading to an increase in the affinity for K^+^ and the Vmax of K^+^ transport [51]. The activity of AKT1 (i.e., OsAKT1 and AtAKT1) is also mediated by phosphorylations [52]. Interestingly, the abundance of protein phosphatase (A0A3B5Y6D9) and HAK (A0A3B6I5B5) increased in the LK-stressed samples. Whether the activation of HAKs in wheat is also dependent on phosphorylations needs to be determined in future investigations.

Ubiquitination is a multi-step process involving the sequential action of E1 (activating enzyme), ubiquitin E2 (conjugating enzyme), and ubiquitin E3 (ligase). Ubiquitin-dependent protein degradation pathways are critical for plant growth and responses to diverse stresses, including drought, high salinity, and nutrient deprivation [53]. The lack of ubiquitin-specific protease16 (AtUBP16) enhances the sensitivity of Arabidopsis to salt because of the associated effects on the Na^+^/H^+^ antiport activity and serine hydroxymethyltransferase stability [54]. Additionally, the up-regulation of E1 was previously observed in alligator weed roots exposed to LK stress [10]. The abundance of a NEDD8-activating enzyme E1 catalytic subunit (A0A3B6EEE4), which is highly similar to Arabidopsis E1 C-terminal related 1 (AtECR1) (i.e., amino acid sequence identity of 70.39%), increased under LK conditions. In Arabidopsis, AtECR1 contains a C-terminal RUB E1 domain and functions as a heterodimer with AXR1 to activate RUB [55]. Our results indicate that phosphorylations and ubiquitinations may be involved in wheat root responses to LK stress.

### 3.4. Transporters and Channel Proteins in Wheat Roots under LK Conditions

Potassium ions can interact with micronutrients, such as iron, manganese, zinc, and copper, leading to decreased or increased nutrient uptake, transport, and utilization [56]. Zinc contributes to the enzymatic catalysis of reactions that require an electrophile, whereas iron, manganese, and copper help mediate redox transformations [57]. Thus, these components are important for respiration, photosynthesis, and other plant metabolic processes. The absorption and transport of metal ions depend on transporters, including the zinc transporter (ZIP), heavy metal ATPase (HMA), and ATP-binding cassette (ABC) transporters [58,59]. The two metal transporters that were down-regulated at 24 h after initiating the LK treatment were identified as a ZIP and HMA. Therefore, the uptake of some metals by wheat roots may be affected by LK conditions. An earlier study indicated that increasing the availability of zinc significantly promotes the uptake of K^+^ by cotton [60]. Moreover, the application of K^+^ can decrease the toxicity of Cd by increasing antioxidant enzyme activities, thereby enhancing faba bean (*Vicia faba* L.) growth [61].

In plants, ABC transporters are involved in the transport and accumulation of substances, stress defenses, and other physiological activities [62,63]. The abundance of a multidrug resistance protein 1 homolog (TaMDR1), which belongs to the ABC transporter family, increased under LK conditions. In wheat, *TaMDR1* expression is induced by aluminum; this induction is caused by the disruption of calcium (Ca^2+^) homeostasis, which occurs soon after exposure to toxic levels of aluminum [64]. Additionally, Ca^2+^ is an important signaling molecule involved in the regulation of K^+^ transport [65]. Intracellular Ca^2+^ levels are modulated as part of the initial response to LK stress [66]. However, the relationship between plant responses to LK stress and micronutrient uptake mediated by these transporters needs to be precisely characterized in future studies.

Aquaporins (AQPs) facilitate the transport of water and other small solutes across cell membranes [67]. A TIP, which belongs to the plant AQP family responsive to abiotic stresses, was up-regulated under LK conditions. The overexpression of *SlTIP2;2* can positively affect plant tolerance to drought, salinity, and cold stress by increasing the osmotic water permeability of the tonoplast and the osmotic buffering capacity of vacuoles [68]. The expression of the wheat *TaTIP2;2* gene negatively regulates drought and salinity stress responses in transgenic Arabidopsis via an abscisic acid-independent pathway [69]. In addition, AtTIP1;1 and AtTIP1;2 can serve as channels for hydrogen peroxide (H_2_O_2_), suggesting they may be associated with stress signaling pathways induced by ROS [70]. Thus, the regulatory effects of TIPs on plant responses to LK stress, as well as the underlying mechanisms, should be investigated.

### 3.5. The K^+^ Transporter TaHAK1-4A Contributes to the K^+^ Uptake by Wheat Roots under LK Conditions

The *HvHAK1* gene, which encodes a high-affinity potassium transporter, was the first KUP/HAK/KT gene in higher plants to be cloned [71]. Since then, various homologs of *HvHAK1* have been identified and characterized, including *AtHAK5*, *OsHAK1*, *OsHAK5*, *ZmHAK5*, and *HbHAK1* [18,19,20,23,72]. The expression levels of these genes are up-regulated significantly under LK conditions as part of a common plant adaptive response to LK stress. Notably, the tissue-specific expression patterns of these genes have been reported. For example, in Arabidopsis, *AtHAK5* is a major transporter mediating the high-affinity uptake of K^+^ by the roots [25]. *ZmHAK5* is mainly expressed in maize roots, wherein it influences K^+^ uptake, whereas *ZmHAK1* is primarily expressed in maize shoots; the overexpression of *ZmHAK1* significantly affects K^+^ distribution in the shoots [23]. *OsHAK5* is highly expressed in the root epidermis and stele, as well as in vascular tissues and mesophyll cells. The encoded protein mediates the uptake of K^+^ by the roots and the transport of K^+^ from the roots to the shoots of LK-stressed rice plants [20]. We identified a K^+^ transporter (TaHAK1-4A) that was up-regulated in the roots exposed to the LK stress. The RNA-seq data revealed that *TaHAK1-4A* was mainly expressed in the roots, implying that it modulates the uptake of K^+^ from the soil.

A yeast complementation assay indicated that TaHAK1-4A mediates K^+^ uptake under extreme LK conditions. On the basis of the analyses of the LK-induced phenotypes and physiological indices of the control and TaHAK1-4A-overexpressed and -silenced plants, we determined that TaHAK1-4A controls the uptake of K^+^ by plants, which is consistent with the induced accumulation of TaHAK1-4A under LK conditions. Furthermore, the overexpression of *TaHAK1-4A* improved the growth of the Arabidopsis *athak5* mutant exposed to the LK stress, implying that TaHAK1-4A and AtHAK5 may have similar functions related to K^+^ uptake by the roots. Our results suggest that TaHAK1-4A is a high-affinity K^+^ transporter involved in the uptake of K^+^ by wheat in response to LK stress.

## 4. Materials and Methods

### 4.1. Wheat Seedling Culture and LK Stress

“KN9204” was used in this investigation, which is a K deprivation-tolerant cultivar selected based on our previous study [8]. Seedlings (12 days old) under full K^+^ condition (6 mM K^+^) were subjected to LK stress with a modified Hoagland solution containing LK (15 μM K^+^). The roots were harvested at 0, 24, and 48 h for further analysis. Three independent biological replicates were conducted per treatment. The root samples of each biological replication were collected from 15 plants and stored at −80 °C.

### 4.2. Protein Extraction

Nine root samples were ground individually into fine powder in liquid nitrogen. The cold acetone method was employed to extract total protein of these samples. The powder of each sample was lysed with protein lysis buffer (pH 8, 6 M Urea, 100 mM NH_4_HCO_3_, and 0.2% SDS), followed by 5 min ultrasonication on ice. The supernatant of lysate was transferred into a new pre-cooled tube after centrifugation. The samples were incubated at 56 °C for 1 h with 10 mM DTT, followed by an alkylation reaction under darkness conditions at room temperature for 1 h. The samples were mixed with pre-cooled acetone and kept at −20 °C overnight. After centrifugation at 12,000× *g* at 4 °C for 15 min, the precipitation was collected. The precipitated samples were washed with pre-cooled acetone three times. The protein samples were redissolved in a dissolution buffer containing 6 M urea, and 100 mM TEAB (pH 8.5). The protein concentration was quantified according to the Bradford assay methods. The gradient concentration of the BSA standard solution ranged from 0 to 0.5 μg/μL.

### 4.3. Digestion and Tandem Mass Tag (TMT) Labeling of Peptides

For digestion treatment, a 120 μg sample was taken and the volume was increased up to 100 μL with lysis buffer; 500 μL of 0.05 M TEAB and 3 μL of 1 μg/μL trypsin were added. Samples were mixed and digested at 37 °C overnight, then an equal volume of 1% formic acid (FA) was added into the samples. After centrifugation at 12,000× *g* for 15 min, the supernatant was injected into the C18 desalting column, followed by washing (three times) and elution (twice). The eluents of each root sample were reconstituted in 100 μL of 0.1 M TEAB and incubated for 2 h at room temperature after mixing with TMT reagent. A common reference was created by pooling an equal quantity of the nine samples. The 0 h_1, 0 h_2, 0 h_3, 24 h_1, 24 h_2, 24 h_3, 48 h_1, 48 h_2, and 48 h_3 samples were labeled with the TMT tags 127N, 127C, 128N, 130N, 130C, 131, 128C, 129C, and 129N, respectively.

### 4.4. High-pH Reversed-Phase HPLC Fractionation and LC-MS/MS Analysis

The samples were fractionated using a water BEH C18 column (5 μm, 4.6 × 250 mm) on a Rigol L3000 HPLC system, the column oven was set as 50 °C. The eluates were monitored at UV 214 nm. We collected the peptides each minute and finally combined them into 10 fractions. The samples were dried under vacuum and then reconstituted with 0.1% (*v*/*v*) FA in water. TMT-labeled samples were analyzed using an EASY-nLCTM 1200 UHPLC system coupled with a Q Exactive HF mass spectrometer operating in the data-dependent acquisition mode. Each sample (1 μg) was injected into a home-made C18 Nano-Trap column (3 μm, 2 cm × 75 μm). Peptides were separated in a home-made analytical column (1.9 μm, 15 cm × 150 μm) using a linear gradient elution with a flow rate of 600 nL/min. The separated peptides were analyzed by Q Exactive HF mass spectrometer (Thermo Fisher). Parameters were set as follows: ion source of Nanospray Flex™ (ESI) spray voltage of 2.3 kV, and ion transport capillary temperature of 320 °C. Full scan range from *m/z* 350 to 1500 was used with a resolution of 60,000 (at *m*/*z* 200), along with an automatic gain control (AGC) target value of 3,000,000 and a maximum ion injection time of 20 ms. The top 20 precursors of the highest abundant in the full scan were selected and fragmented by higher energy collisional dissociation (HCD) and then analyzed in MS/MS, where the resolution was 15,000 (at *m*/*z* 200) for 6 plex. The AGC target value was 50,000 and the maximum ion injection time was 45 ms. The normalized collision energy was set as 32%, the intensity threshold was set as 1,900,000, and the dynamic exclusion parameter was 20 s.

### 4.5. Bioinformatics Analysis of Identified Proteins

Proteome Discoverer v2.2 software was used to analyze the raw data. The reference protein database was downloaded from the Uniport database (https://www.uniprot.org/, accessed on 25 October 2019). Trypsin was specified as a cleavage enzyme. A maximum of two miscleavage sites were allowed. The mass tolerance for the precursor ion was 10 ppm and mass tolerance for product ion was 0.02 Da. Carbamidomethyl was specified in PD 2.2 as fixed modifications. Oxidation of methionine (M), acetylation of the N-terminus, and TMT 6-plex of tyrosine and lysine were specified in PD 2.2 as variable modifications. The identified proteins with|fold changes| > 1.20 and *p*-value < 0.05 in different compared groups were considered as differential abundance proteins (DAPs).

The Cluster of Orthologous Groups (COG) of proteins was analyzed with the database from NCBI (http://www.ncbi.nlm.nih.gov/COG/, accessed on 10 February 2020). The Gene Ontology (GO) was analyzed with the Enrich Pipeline process [73]. The PPI networks of proteins were analyzed using the String database (http://string-db.org/, accessed on 21 February 2020).

### 4.6. Identification of HAK Gene Family and Expression Profiles of HAKs under the LK Stress in Wheat

The identification of the *TaHAK* gene family (Pfam ID: PF02705) was performed according to the method in our previous study [74]. The reference wheat genome was assembly version IWGSC 2.0, downloaded from Ensembl Plants database (http://ftp.ensemblgenomes.org/pub/plants/release-51/fasta/triticum_aestivum/, accessed on 21 November 2021). The phylogenetic tree was constructed using the neighbor-joining (NJ) method in MEGA X, with 1000 bootstrap replicates. The expression patterns of *TaHAKs* under the LK stress in wheat were analyzed based on our previous RNA-Seq data [8]. The expression analysis of *TaHAK1-4A* in different tissues was based on the publicly available wheat RNA-Seq datasets (http://bar.utoronto.ca/efp_wheat/cgi-bin/efpWeb.cgi, accessed on 21 November 2021) [27].

### 4.7. RNA Extraction and Transcript Analysis

Total RNA was extracted from seedling roots with the FastPure Plant Total RNA Isolation Kit (Vazyme, Nanjing, China) following the manufacturer’s instructions. The first-stand cDNA was synthesized using five HiScript^®^III qRT SuperMix Kits (Vazyme, Nanjing, China). RT-PCR was performed with the ChamQ Universal SYBR qPCR Master Mix (Vazyme, Nanjing, China) and the QuantStudio 5 Real-time PCR system (Applied Biosystems, Waltham, MA, USA). TaTEF1 and AtTUB2 served as constitutive genes in wheat and Arabidopsis, respectively. All reactions were run in triplicate to obtain comparable results. Statistical analysis for RT-PCR data was performed using the 2^−ΔΔCt^ method. Specific primers used for RT-PCR are shown in Table S4.

### 4.8. Functional Complementation of TaHAK1-4A in Yeast

The coding sequence (CDS) of *TaHAK1-4A* was cloned into the p416-GPD vector driven by the GAP promoter, then transformed into the K^+^ uptake-deficient strain R5421 (*trk1∆*, *trk1∆*), in which *TRK1* and *TRK2* (two endogenous K^+^ transporter genes) were deleted. R757 was used as a positive control. The yeast complementation experiment was performed according to the method described by Li et al. [52]. In brief, p416-GPD and p416-TaHAK1-4A were transformed into R5421 and named p416-GPD and TaHAK1-4A, respectively. R5421 and R757 were grown on arginine phosphate (AP) medium supplemented with different concentrations of K^+^.

### 4.9. Subcellular Localization of TaHAK1-4A

The CDS, excluding the stop codon of *TaHAK1-4A*, was obtained via PCR amplification using the specific primers (Table S4) and inserted into the pSuper1300-GFP vector containing the MAS promoter (MAS::GFP). The control plasmid MAS::GFP and the fusion plasmid MAS::TaHAK1-4A-GFP were introduced into *Agrobacterium tumefaciens* strain GV3101, which was then transformed into epidermal cells of *N. benthamiana*. The tobacco plants were cultivated for 48 h in darkness. Green fluorescent protein (GFP) and fluorescence signals were observed using confocal laser scanning microscopy.

### 4.10. Generation of Arabidopsis Transgenic Plants and LK Stress Response Analysis

The CDS of *TaHAK1-4A* was inserted into pCAMBIA-1302 with the CaMV 35S promoter (35S::*TaHAK1-4A*). The 35S::*TaHAK1-4A* was transformed into *Agrobacterium* strain GV3101 and introduced into the *athak5* mutant or the WT Arabidopsis using the floral dip method [75]. Seeds of transgenic plants were selected on MS medium with hygromycin. Arabidopsis plants were grown in pots filled with vermiculite and kept in growth chambers at 23 °C under 16 h light/8 h darkness for seed collection. For the phenotype assay of plants, seeds were placed on different K^+^ containing media (10 µM and 5 mM) and treated at 4 °C for 3 d in darkness, and then the plants were grown at 23 °C under 16 h light/8 h darkness for 14 d in a growth chamber.

### 4.11. Silencing of TaHAK1-4A and LK Stress Response Assay in Wheat

Silencing of *TaHAK1-4A* with barley stripe mosaic virus (BSMV) was performed according to the method from our previous study [30]. The in vitro-synthesized BSMV RNA of BSMV:*α*, BSMV:*β*, BSMV:*γ0,* or BSMV:*target* (BSMV:*TaHAK1-4A* and BSMV:*PDS*) was inoculated on the first fully expanded leaves of 8 d old“KN9204” wheat by rub-inoculation with a gloved finger. The seedlings were treated in a dark, damp incubator for 12 h and then were transferred to the modified Hoagland solution with full K^+^ (6 mM K^+^) or LK (15 µM K^+^) at 16/8 h light/dark at 23 °C. Wheat plants infected with BSMV:*γ0* and BSMV:*PDS* were used as the control and positive control, respectively. After 12 days of BSMV inoculation, the third leaf of each plant was collected for expression analysis of *TaHAK1-4A* using RT-PCR (Table S4). The fresh weight, dry weight, length, and K^+^ concentration of seedlings infected with BSMV:*γ0* or BSMV*:TaHAK1-4A* were measured as described in our previous study [76]. Net K^+^ flux was measured from root tips using non-invasive micro-test technology (NMT) according to the method described by Zhang et al. [77].

### 4.12. Statistical Analysis

SPSS v25 statistical software (IBM Corp., Armonk, NY, USA) was used for the data analysis. Student’s *t*-test was used to test the statistical significance between the control and treatment. GraphPad Prism 8 (https://www.graphpad-prism.cn/, accessed on 25 November 2021) was used for the histogram drawing. Each value is the mean standard deviation from at least three independent biological replicates.

## 5. Conclusions

We performed a TMT-based proteomic analysis that revealed 104 DAPs in wheat roots treated with LK stress. The DAPs were related to carbohydrate and energy metabolism, transport, stress responses and defense, and post-translational modifications induced by LK conditions. The expression of TaHAK1-4A, encoding a high-affinity potassium transporter, was mainly induced by LK stress in wheat roots. Functional analyses of TaHAK1-4A in yeast, Arabidopsis, and wheat indicated that it mediates K^+^ uptake under LK conditions. This study provides insights into the protein network involved in LK stress responses. Furthermore, the identified candidate gene may be useful for enhancing K^+^ contents in wheat plants.

## Supplementary Materials

The following supporting information can be downloaded at: https://www.mdpi.com/article/10.3390/ijms232113504/s1.
