# Supplementary material for: Proteomic Analysis of Roots Response to Potassium Deficiency and the Effect of TaHAK1-4A on K+ Uptake in Wheat
Source: Int J Mol Sci. 2022 Nov 4;23(21):13504. doi: 10.3390/ijms232113504 (PMC9659051; doi:10.3390/ijms232113504)
Supplement: Supplementary file 1 [file ijms-23-13504-s001.zip › Supplementary_Figures.pdf]

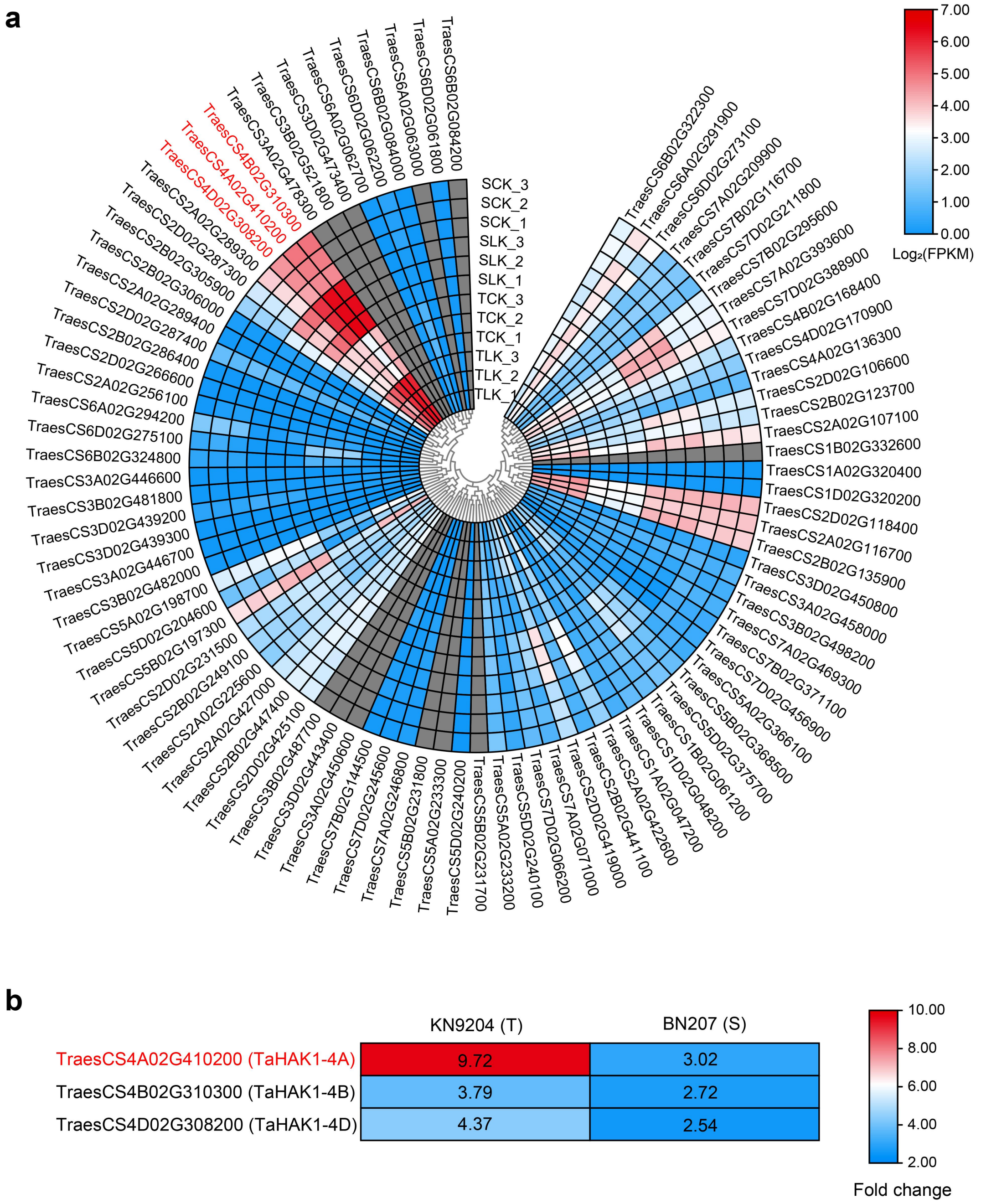

Figure S1. Expression profiles of *TaHAKs* in wheat roots under CK and LK conditions. (**a**) Hierachical clustering and expression patterns of 89 *TaHAKs* in wheat roots. (**b**) Fold change of *TaHAK1-4A*, *TaHAK1-4B* and *TaHAK1-4D* in 'KN9204' and 'BN207' after LK stress. Wheat varieties 'BN207' (low K^+ sensitive) and 'KN9204' (low K^+ tolerant) were abbreviated to S and T, respectively (e.g., BN207 under control was named as SCK).

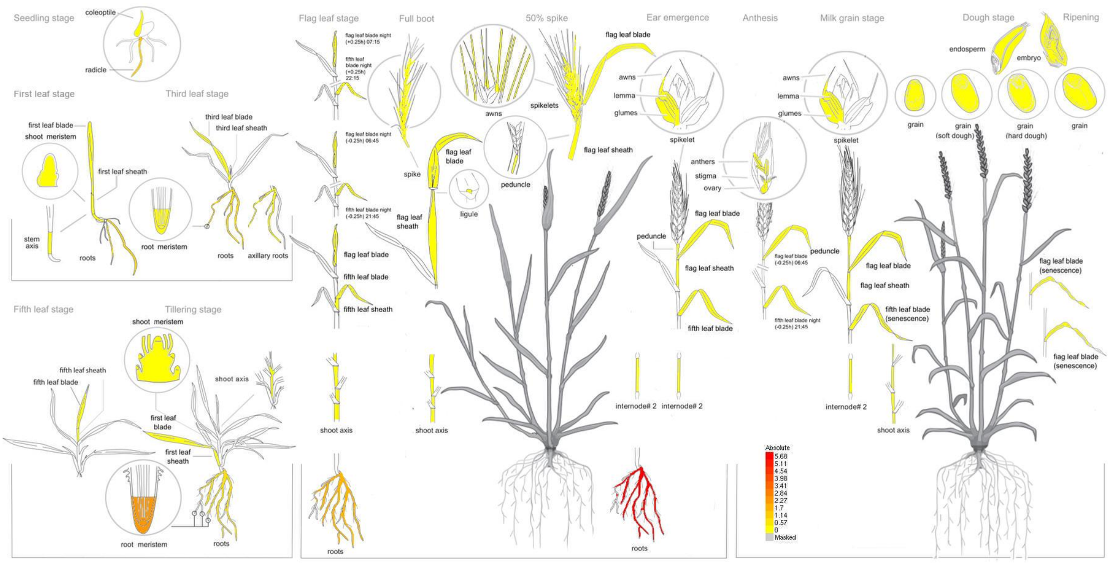

Figure S2. Expression level analysis of *TaHAK1-4A* in various development stages. Data were downloaded from http://bar.utoronto.ca/efp_wheat/cgi-bin/efpWeb.cgi. Expression levels were summarized as TPM from the transcript to the gene level using tximport v1.2.0.
